# Effects of Ginger and Expectations on Symptoms of Nausea in a Balanced Placebo Design

**DOI:** 10.1371/journal.pone.0049031

**Published:** 2012-11-13

**Authors:** Katja Weimer, Jörg Schulte, Annamaria Maichle, Eric R. Muth, Jenna L. Scisco, Björn Horing, Paul Enck, Sibylle Klosterhalfen

**Affiliations:** 1 Department of Psychosomatic Medicine and Psychotherapy, University Hospital Tübingen, Tübingen, Germany; 2 Department of Psychology, Clemson University, Clemson, South Carolina, United States of America; The University of Hong Kong, Hong Kong

## Abstract

**Objective:**

Ginger effects on (experimental) nausea have been described, but also strong placebo effects and sex differences when nausea is involved. The “balanced placebo design” has been proposed to allow better separation of drug and placebo effects.

**Methods:**

Sixty-four healthy participants (32 women) were randomly assigned to receive an antiemetic ginger preparation or placebo, and half of each group was told to have received drug or placebo. They were exposed to 5×2 min body rotations to induce nausea. Subjective symptoms and behavioral (rotation tolerance, head movements) and physiological measures (electrogastrogram, cortisol) were recorded. Groups were balanced for sex of participants and experimenters.

**Results:**

Ginger and the information given did not affect any outcome measure, and previous sex differences could not be confirmed. Adding the experimenters revealed a significant four-factorial interaction on behavioral but not on subjective or physiological measures Men who received placebo responded to placebo information when provided by the male experimenter, and to ginger information when provided by the female experimenter. This effect was not significant in women.

**Conclusion:**

The effects of an antiemetic drug and provided information interact with psychosocial variables of participants and experimenters in reports of nausea.

## Introduction

Herbal and alternative medicine remedies such as ginger [1] are widely used and accepted [2] for the treatment of the various clinical conditions associated with nausea, e.g. in motion and sea sickness [3], chemotherapy-induced nausea [4], and pregnancy [5]; however, efficacy data have remained controversial [6].

Placebo responses have often been recognized when nausea was treated with drugs [7] and with non-pharmacological treatments such as acupressure [8] and acupuncture [9]. Chemotherapy-induced nausea and vomiting is also known for strong placebo effects in clinical trials [10], and “anticipatory nausea” in cancer treatment [11] that occurs following repetitive chemotherapy exposure is probably the best-documented clinical example of a Pavlovian conditioning procedure [12]. Pavlovian conditioning, on the other hand, is thought to be one major underlying psychobiological mechanism for placebo responses in medicine [13].

We have previously shown that sex differences in overall susceptibility to (motion-induced) nausea are well established [14], that nausea symptoms can be readily evoked following a Pavlovian conditioning procedure in healthy participants [15] and in patients [16], and that this is more effective in females than in males [17]. In contrast, males responded stronger to suggested symptom worsening (“nocebo responses”) not only in a nausea paradigm [18] but also in pain and placebo analgesia experiments [19].

In these experiments, we were so far unsuccessful eliciting symptom improvements (placebo responses), while in contrast symptom worsening (nocebo responses) were much easier to provoke. This may have in part be due to the fact that we selected participants susceptible to motion that have a history of motion sickness symptoms in their past, and such participants may be less likely to develop expectancies of improvement with strong nauseogenic stimuli. In fact there is some indication in the literature that placebo suggestions may produce opposite effects due to disappointment when the stimulus onset indicates that the applied drug may not be sufficient to suppress intestinal symptoms [20].

The balanced placebo design [21] has been proposed to differentiate between the true drug and placebo effects in comparison to the confounding of drug and placebo effects in drug arms of randomized placebo-controlled drug trials [22].

With this study we aim to determine whether a) ginger has an antiemetic effect in our nausea paradigm (rotation-induced motion sickness), b) whether information provided on having received ginger or placebo prior to testing would affect ginger-modulated nausea, and c) whether there is a difference in subjective symptoms, behavioral measures and objective assessment of physiological (gastric) functions. We finally wanted to replicate previous gender effects in rotation tolerance.

## Methods

### Participants

We recruited 64 healthy male and female participants (24.3±3.2 years, 20–38 years, 32 women) from the student population of the University of Tübingen, Germany. All participants were naïve to the rotation procedure and had not participated in a previous experiment regarding nausea, motion sickness and/or placebo effects. Women were scheduled during the luteal phase of their menstrual cycle.

Participants selected were interviewed by one of two experimenters (AM, JS) using a routine anamnesis tool to exclude concomitant medication (except contraceptives), acute and chronic diseases of the central nervous system, the gastrointestinal system, and other chronic conditions. They were informed about the purpose of the study as testing the effects of a herbal remedy, ginger, on motion sickness symptoms in a double-blinded, randomized and placebo-controlled fashion.

### Ethics Statement

The study protocol was approved by the Ethical Review Board of the University Medical School Tübingen, and participants gave written informed consent prior to inclusion. Complete disclosure of the study purpose was offered to all participants after completion of the study.

### Study Design

After written consent, participants were randomly assigned to one of four groups according to the “balanced placebo design” [21] (Table 1): Half of the participants were assigned to receive 1 g of ginger (powder in 3 gelatin capsules) (Zintona®, Grünwalder GmbH, Bad Tölz, Germany) while the other half would receive equally looking capsules containing a placebo (1 g of starch). One hour after intake of the capsules and immediately prior to rotation half of each group was informed they had received ginger, while the other half was informed they had received placebo. This distribution was balanced for sex and experimenter.

**Table 1:** The balanced placebo design.

		Information	
		Drug	Placebo
**Application**	**Drug**	true positive	false negative
	**Placebo**	false positive	true negative

Prior to testing, participants were asked to score their susceptibility to rotation stimuli (expectancy value 1, EV 1) and whether they believe that ginger would affect their nausea during rotation (EV 2) on a 0 to 100 visual analog scale (VAS).

### Experimental Procedure

Participants were scheduled to come to the laboratory on a single day in the morning at 8.00 or 10.30 a.m. and to maintain fasted for the last 6 hours prior to the test. Compliance with the fasting instruction was controlled with a glucose stick.

After placing cutaneous electrodes for the electrogastrogram (EGG) on the surface of their skin above the stomach (see below), participants were seated in a rotation chair as previously described [15], [18]. After 15 min of baseline EGG recording, they took the ginger/placebo capsules followed by another 15 min of EGG recording. For the next 45 min participants filled out questionnaires (Motion Sickness Susceptibility Questionnaire (MSSQ) [23]) with continued EGG recording before the rotation procedure started. Immediately prior to the rotation procedure, participants were informed about the received medication according to the balanced placebo design (see Table 1).

Immediately before rotation participants were blindfolded and rotated in a standardized fashion for 5 runs of 2-minute duration each with 1-minute interruptions in-between as described previously [15]. Rotation speed was set at 120 degrees/sec, and participants were instructed by a loudspeaker to move their head up or down every 10 sec, as previously described [18]. Participants could interrupt the head movements if nausea occurred, and they could stop the rotation procedure entirely if severe nausea occurred, but were encouraged to continue the next run until 5 runs had at least been started. Head movements are essential for the development of the so-called Coriolis effect during which participants experience an illusionary tumbling movement of their body that leads to nausea symptoms [24]. If head movements are stopped symptoms do not increase further. Therefore, the number of head movements (HM) performed as well as the total rotation time (RT) were noted as separate behavioral outcome measures.

Prior to the rotation, at each interval between two runs, immediately after termination and 15 minutes following termination symptom ratings (between 0 = none and 5 = maximal) were taken on a 7-item symptom list asking for the presence or absence of nausea-associated symptoms (vertigo, headache, nausea, urge to vomit, tiredness, sweating, stomach awareness), as previously described [15], [18]. They were used to calculate symptom scores, ranging between 0 and 35 for baseline (SR0), the maximum symptom scores during the rotation (SRmax), and the post-rotation score (SR15).

**Figure 1:**
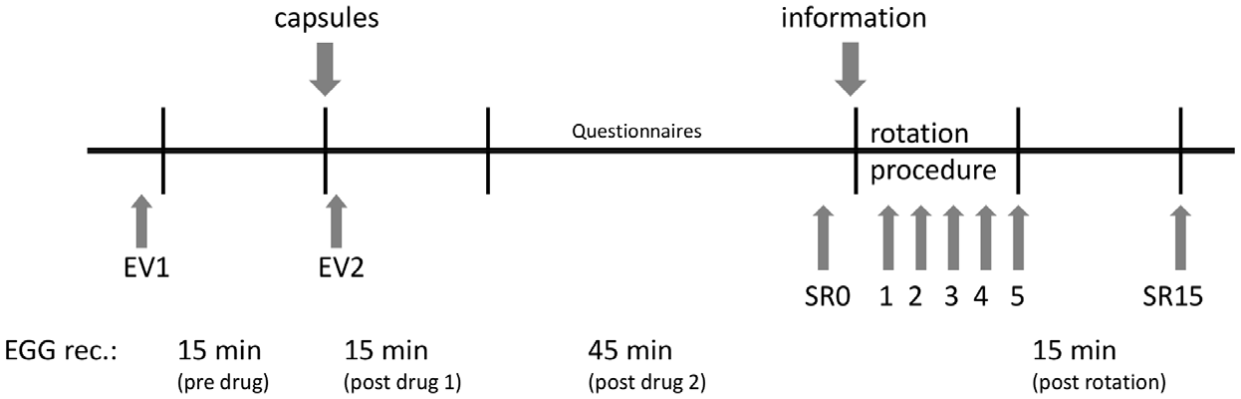
Time schedule of the single session of the study in an individual participant.

At the beginning of the experiment, immediately before rotation, after rotation termination and 15 min following rotation termination saliva samples were taken for analysis of saliva cortisol levels as previously described [25]. EGG was recorded for 15 min after the termination of the rotation procedure. The time course of the entire experiment is illustrated in Figure 1.

All interviews and investigations were conducted by one of two experimenters, a male and a female (JS, AM), and each of them investigated half of the participants in each of the four groups, again balanced for sex of the volunteers.

### Electrogastrogram

Gastric myoelectrical activity was recorded by an electrogastrogram (EGG) for which three skin electrodes were placed above the stomach as described in the literature [8] and connected to a Biolog device with Fetrodes technology (UFI, Morrow Bay, CA, USA). The EGG was recorded with a sampling rate of 5 Hz (Filter settings: band-pass filter with a low cutoff of 0.014 Hz and a high cutoff of 0.34 Hz both with 12 dB per oct roll-off) and stored for off-line analysis.

Recordings were screened visually for artifacts. Criteria for artifacts were signals with improbable amplitudes (+/−1000 µV) for myoelectrical activity of the stomach and fast and sudden onset that did not fit to the surrounding signals. Three segments of at least 5 min length (pre drug, post drug 1, post drug 2) from baseline recordings, and one from the post rotation period (post rotation) were selected for analysis (see Figure 1). Selected EGG data were analyzed with a Fast Fourier Transformation procedure (FFT) (custom software using Prime Factor FFT for Windows, version 3.03, Alligator Technologies, Costa Mesa, CA, USA) and a spectral resolution of 0.25 cycles per minute (cpm). A frequency range between 2.5 to 3.75 cpm was regarded as normal gastric activity (normogastria) and a range between 4.0 to 9.75 cpm as tachygastria. We then calculated the percentage spectral power from the total range of 0.75 to 15.0 cpm (for detailed information see [26]) and computed the ratio between the percentage of the normogastria and the tachygastria band as indicator for nausea. Ratio values above 1 indicate normal gastric activity and values below 1 indicate increased tachygastria. The interruption of the normal 3 cpm activity of the stomach and a shift towards tachygastria has been repeatedly associated with nausea, e.g. induced by rotation chair or vection drum [8], [27].

### Statistics

Equal distribution of demographics and other characteristics among the four experimental groups prior to intervention (baseline data) were tested by 2×2 ANOVAs and Chi-Square tests (Table 2). To assess whether ginger exhibited a nausea-reducing effect at all with our rotation procedure and whether the instructions given produced an effect on nausea, participants were compared using a 2×2 factorial multivariate analysis of variances (MANOVA) with the between factors drug (ginger, placebo) and information (ginger, placebo) and tested for effects on SRmax, RT and HM. Additionally, sex of participants as well as the experimenters were included into 2×2×2 and 2×2×2×2 MANOVAs, respectively. Because of the difficulty in interpreting significant interactions in multi-factorial MANOVAs, we subsequently analyzed data separate for sex of participants.

**Table 2:** Baseline data prior to interventions in experimental groups.

Drug	Placebo	Placebo	Ginger	Ginger	
Info	Placebo	Ginger	Placebo	Ginger	Statistics
Sex (female:male)	8∶8	8∶8	8∶8	8∶8	n.s.
Age	23.4±2.8	24.9±4.2	24.6±3.4	23.1±2.0	n.s.
MSSQ^1^	40.2±31.9	37.6±22.2	41.0±25.0	46.6±18.4	n.s.
Expectancy 1^2^	26.8±17.8	45.0±21.5	50.7±18.8	36.9±12.9	p = .001
Expectancy 2^3^	28.4±16.5	40.2±20.6	46.0±21.3	31.1±11.2	p = .004
Cortisol 1^4^	19.0±12.5	22.1±15.5	17.3±10.9	20.1±11.8	n.s.
Cortisol 2^5^	10.9±6.2	12.4±9.0	10.3±9.3	10.4±7.7	n.s.
SR0^6^	1.69±1.1	3.06±2.3	1.75±1.9	2.50±2.3	n.s.
EGG a^7^: n	16	16	16	15	n.s.
EGG a: 3 cpm (%)^8^	29.3±12.5	27.6±12.0	26.2±8.7	28.7±13.8	n.s.
EGG a: Tachy (%)^9^	21.3±6.3	22.5±7.5	23.0±6.6	19.4±4.4	n.s.
EGG a: Ratio^10^	1.49±0.77	1.37±0.73	1.26±0.72	1.57±0.89	n.s.
EGG b^11^: n	12	14	13	13	n.s.
EGG b: 3 cpm (%)^8^	28.6±11.8	28.8±12.4	26.5±7.3	26.9±9.6	n.s.
EGG b: Tachy (%)^9^	20.3±6.4	20.6±5.4	23.7±6.7	19.5±4.6	n.s.
EGG b: Ratio^10^	1.52±0.73	1.49±0.70	1.18±0.37	1.44±0.53	n.s.

1 Motion Sickness Susceptibility Questionnaire score;

2 Expectancy of susceptibility to rotation stimuli (VAS);

3 Ginger expectancy value (VAS) prior to rotations;

4 in the morning upon arrival in the lab;

5 immediately prior to rotation;

6 Symptom rating before rotations;

7 EGG a: available data at baseline (n = 63);

8 percentage of normal gastric activity;

9 percentage of tachygastria;

10 ratio between normal activity and tachygastria;

11 EGG b: data of cases with all 4 measures (n = 52).

Potential covariates were baseline saliva cortisol levels, expectancy values of symptoms (EV 1) and of ginger effects (EV 2), and scores in the questionnaire (MSSQ).

For the analysis of the EGG data, we used the same factors in repeated measures ANOVAs with the four recording periods (pre drug, post drug 1, post drug 2, post rotation) and the ratio between normal and tachygastric activity as described above.

The significance level was set to 0.05. All analyses were performed with the SPSS Version 13 statistical package.

## Results

### Baseline Measures Prior to Interventions

Table 2 lists demographic and other characteristics of participants as well as measures prior to interventions of the four experimental groups. As can be seen, none of these baseline values were different between the four groups, except the expectancy values (EV1: F(1,60) = 12.614, p = .001; EV2: F (1,60) = 8.907, p = .004).

For the EGG analysis, baseline data of 63 participants were useable, and for all time points the data of 52 participants were useable. Drop-outs due to movement artifacts in the signal were distributed equally across the four groups (Chi-square n.s.).

### Effects of Ginger and Information

Table 3 lists results during and after rotations: When the four groups were compared with respect to the effect of ginger and the information provided, no main effects of ginger or of information, and no interaction between both were found for SRmax, HM and RT (2×2 MANOVA: F(3,58) = 0.358, p = .784; 2×2 ANOVAs: F(1,60) = 0.249, p = .620; F(1,60) = 0.355, p = .554 and F(1,60) = 0.319, p = .574, resp.).

**Table 3:** During and post rotations data between groups.

Drug	Placebo	Placebo	Ginger	Ginger	
Info	Placebo	Ginger	Placebo	Ginger	Statistics
HM	46.1±17.2	48.1±13.9	38.3±19.0	45.1±13.9	n.s.
RT (sec)	497±163	482±142	436±182	466±152	n.s.
SRmax	20.2±5.8	20.5±6.3	18.9±6.8	20.8±6.1	n.s.
Cortisol^1^	25.5±20.0	26.0±17.0	24.0±17.1	27.6±21.1	n.s.
EGG: n	12	14	13	13	n.s.
EGG: 3cpm(%)^2^	16.8±5.5	18.2±8.3	21.7±7.2	20.8±9.3	n.s.
EGG: Tachy(%)^3^	25.8±9.2	23.6±7.7	27.9±7.9	24.0±6.9	n.s.
EGG: Ratio^4^	0.78±0.45	0.92±0.63	0.90±0.59	0.96±0.64	n.s.
EGGunavailable	4	2	3	3	n.s.
Vomiting^5^	3	3	2	5	n.s.
MSSQ(vomited)	54.8±48.4	60.0±16.5	40.8±5.4	53.9±16.8	n.s.
SRmax(vomited)	23.0±3.6	22.0±4.4	23.5±6.4	24.8±3.5	n.s.

HM = head movements, RT = rotation tolerance, SRmax = maximum symptom rating during rotations.

1 cortisol increase with rotation;

2 percentage of normal gastric activity;

3 percentage of tachygastria;

4 ratio between normal activity and tachygastria;

5 number of participants experiencing vomiting after which rotation was terminated (chi-square).

Controlling for the differences in baseline expectancy values did not change the reported results, and including the MSSQ or baseline cortisol as covariates in the analysis separately revealed that none was significant in the MANOVAs and ANOVAs, except the MSSQ that affected SRmax (F(1,59) = 6.376, p = .014) but with no change of the result.

### Participants who Prematurely Interrupted

Of all 64 participants, 13 (8 females) did show symptoms of vomiting and prematurely interrupted the rotation procedure. They were distributed equally across the four groups (Chi-square n.s., see Table 3) and did not significantly differ from the other participants in any of the baseline measures (Table 2) or the outcome measures (Table 3) (t-tests n.s., data not shown), except significantly higher MSSQ scores (t(62) = −2.051, p = .044) and more reported symptoms (t(62) = −2.328, p = .023). If those subjects are excluded from analysis, reported group comparisons for SRmax, HM and RT remain insignificant and controlling for covariates had no influence on these results (data not shown).

### Sex of Participants

Adding participant’s sex as a factor to the MANOVA, the results remained insignificant for all main effects and interactions (MANOVA: F(3,54) = 0.409, p = .747).

Post-hoc separate analyses for female and male participants revealed different relations between outcome measures: Although symptom rating (SRmax) was associated with behavioral measures (RT, HM) in women (r = −.524, p<.001 and r = −.465, p = .002, resp.) these associations were not significant in men (r = −.265, p = .143 and r = −.315, p = .079, resp.). Statistics of baseline measures (as listed in Table 2) did not markedly change when analyzed separate for participant’s sex (Chi-square n.s.). There were still no observed effects of ginger and information or their interaction in the 2×2 MANOVA for HM, RT and SRmax in both male and female participants separately. Controlling for covariates had no influence on any results.

### Effects of Experimenters

For post-hoc analyses of possible experimenter effects, the variable “experimenter” was added into the “drug×information×particpant’s sex” MANOVA: This interaction was significant (F(3,46) = 4.080, p = .012) but only for HM and RT (F(1,48) = 11.433, p = .001 and F(1,48) = 4.191, p = .046).

**Figure 2:**
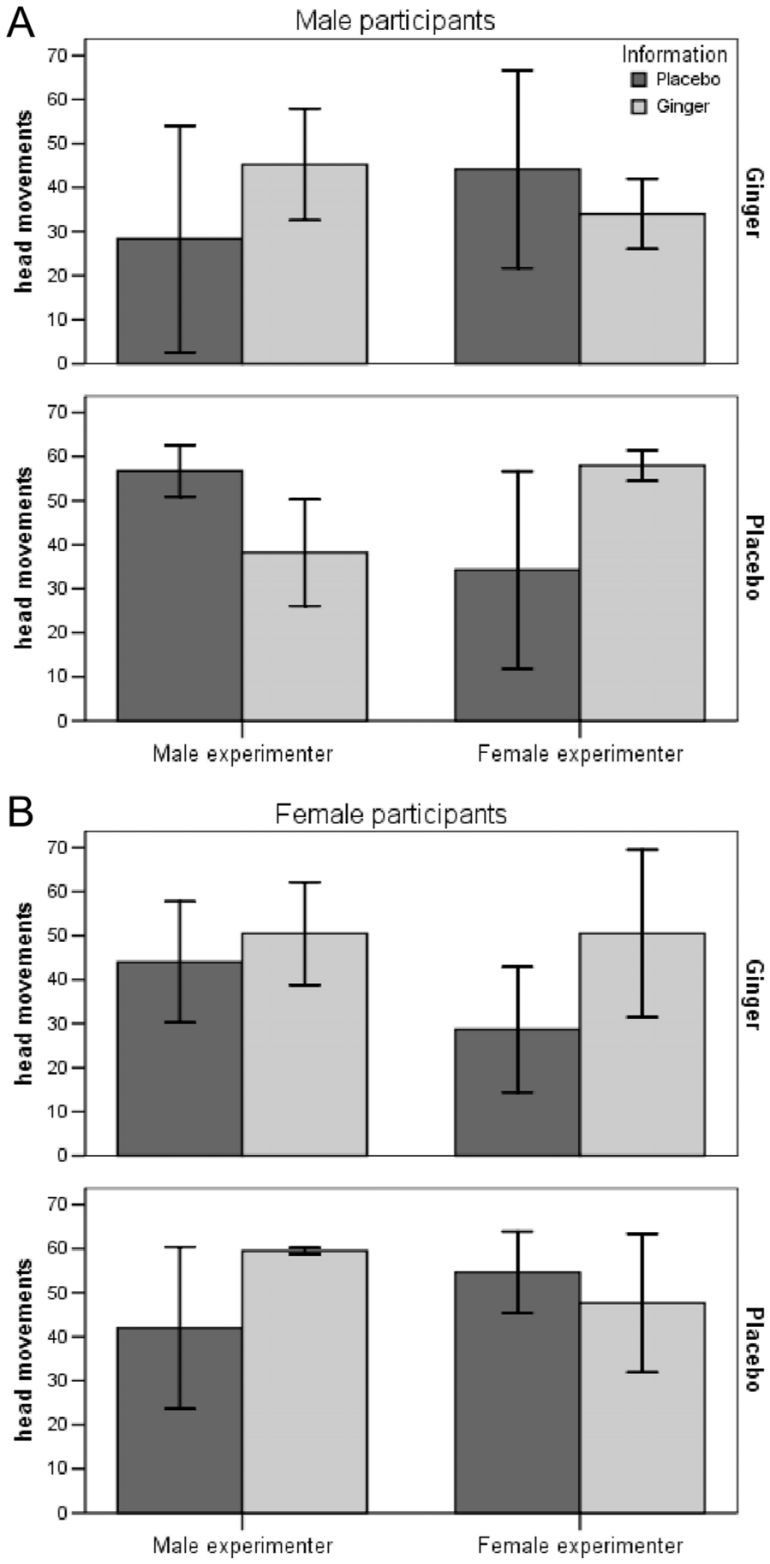
Number of head movements in male and female participants (HM; means +/− SD). Male (Panel A) and female (Panel B) participants received either ginger or placebo in a double-blinded design and (immediately prior to rotation) were informed to have received ginger or placebo in a balanced placebo-design, i.e. half of the participants of each group were correctly informed while the other half received false information. When the four groups were compared by effects of drug and information on symptom rating (SR), rotation tolerance (RT), and head movements (HM), MANOVA results were only significant when sex of participants and the experimenters were added as between factors to the analysis (F = 4.307, p = .009).

Because four-factorial ANOVAs and MANOVAs are difficult to interpret and present, we again performed post-hoc separate analyses for male and female participants. For male participants, the 2×2×2 MANOVA was significant (F(3,22) = 3.344, p = .038) and specifically for HM (F(1,24) = 8.979, p = .006) (Figure 2A). In contrast, in women no multivariate effect was found (F(3,22) = 1.835, p = .170) (Figure 2B).

As Figure 2A shows there was no difference in HM when male participants received ginger (top panel). When they received a placebo (bottom panel) head movements were higher with the placebo information when this information was provided by the male experimenter and with the ginger information when it was given by the female experimenter. Figure 2B showed nearly the opposite response in females, but this interaction was not significant.

None of these results did change when controlling for MSSQ, baseline cortisol, or expectancy values.

### Gastric Activity

**Figure 3:**
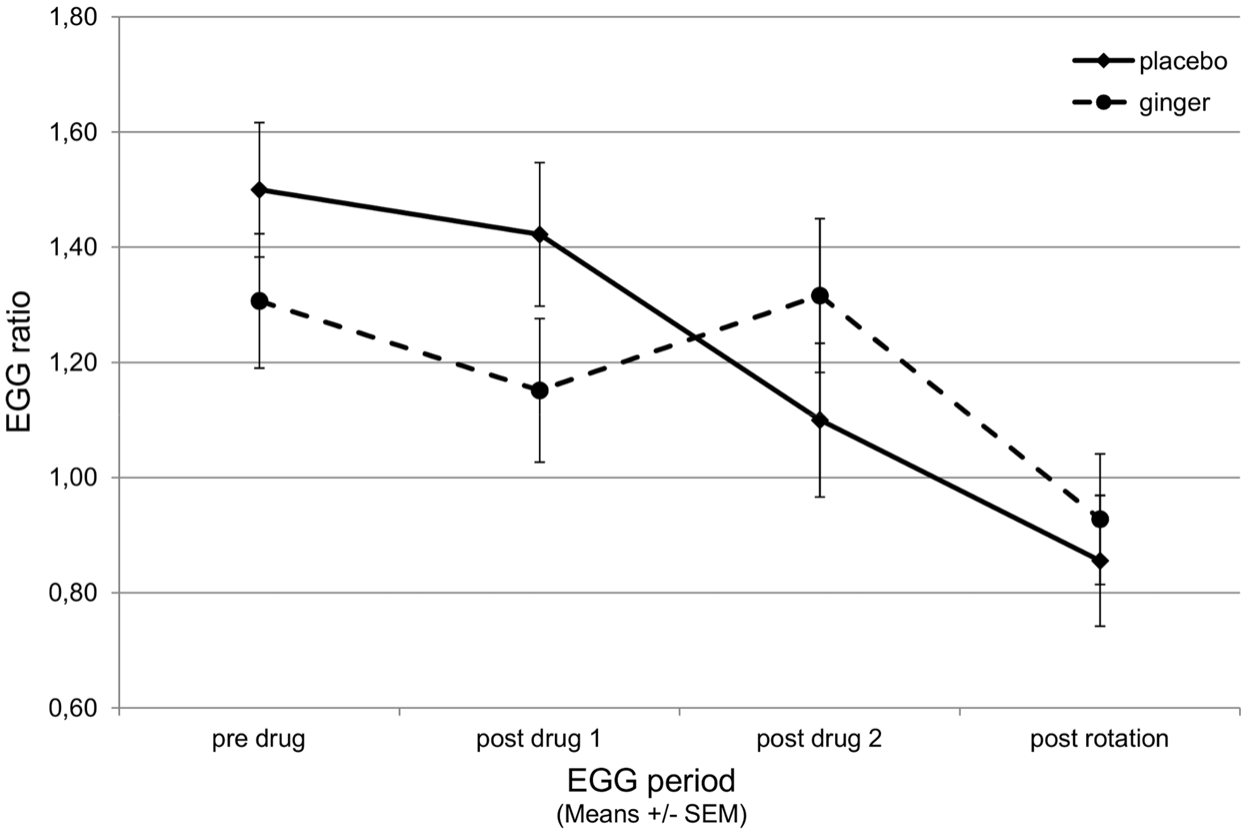
Electrogastrogram (EGG) in participants that received ginger or placebo. EGG was evaluated as the ratio between normal activity (2.5 to 3.75 cycles per minute, cpm) and activity in the tachygastria band (4 to 9.75 cpm), and with increasing nausea the ratio usually falls below 1. Data segments were recorded at baseline, twice after drug application, and after rotation. The constant fall of the ratio from baseline to post rotation is interrupted in the ginger group but ginger was not able to prevent nausea to occur with rotation.

Repeated measures ANOVA of the ratio between normal and tachygastric activity did not show effects of ginger or information or their interaction (F(3,144) = 0.644, p = .588) but there was a significant decrease of the ratio over the 4 periods (F(3,144) = 8.207, p<.001) and a trend towards significance of ginger (F(3,144) = 2.156, p = .096) illustrated in an interruption of the decrease at post-drug2 as seen in the placebo condition (Figure 3). Baseline cortisol values as covariates were not significant.

**Figure 4:**
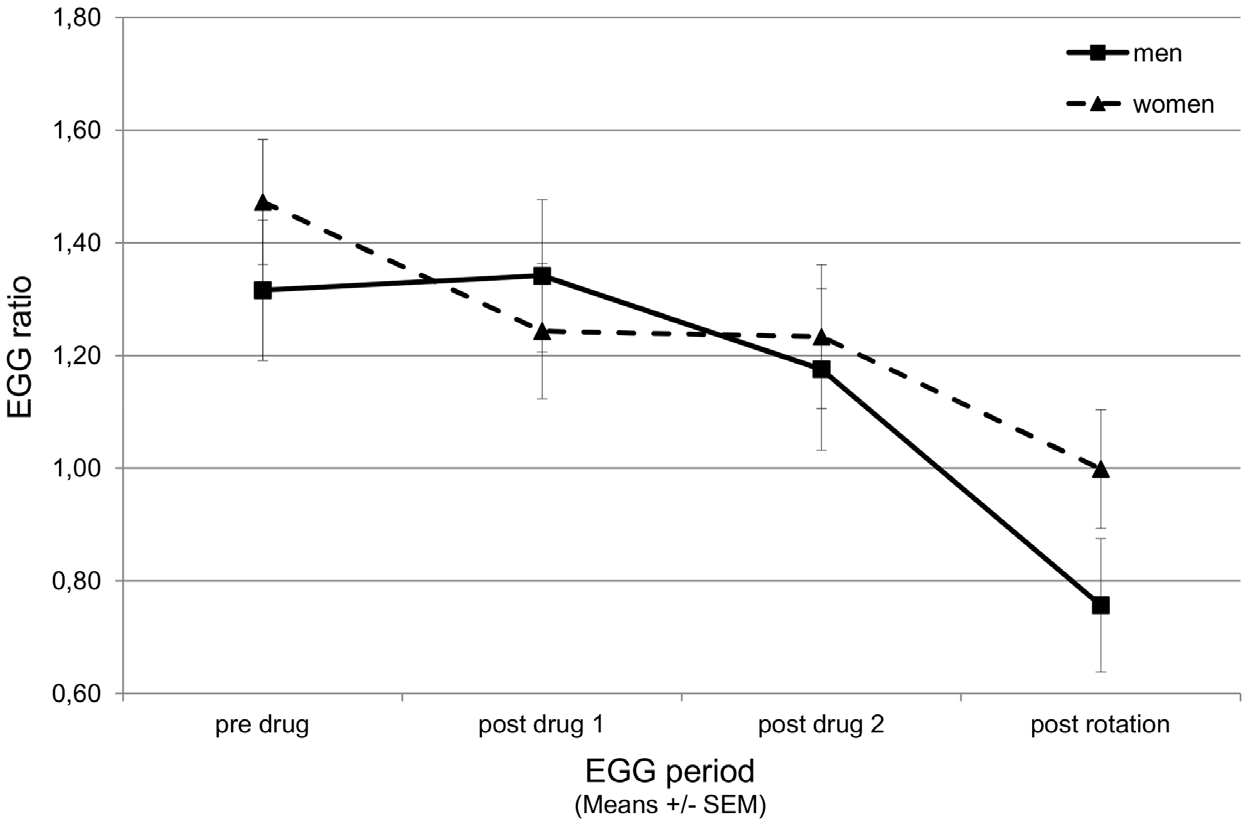
Electrogastrogram (EGG) in male and female participants that received ginger or placebo. EGG was evaluated as the ratio between normal activity (2.5 to 3.75 cycles per minute, cpm) and activity in the tachygastria band (4 to 9.75 cpm), and with increasing nausea the ratio usually falls below 1. Data segments were recorded at baseline, twice after drug application, and after rotation. The constant fall of the ratio from baseline to post rotation was not different between men and women.

In separate repeated measure ANOVAs for female and male participants, the interaction of drug by information was still not significant (F(3,75) = 1.650, p = .185 and F(3,57) = 0.497, p = .686, resp.) but the trend towards significance of drug was only seen in men (F(3,57) = 2.500, p = .069; women: F(3,75) = 1.025, p = .386). Post-hoc analysis with drug as the only between-subjects factor was significant in men (F(3,63) = 2.769, p = .049) but not in women (F(3,81) = 1.007, p = .394) and the effect of time was significant in both women and men separately (F(3,81) = 3.074, p = .032 and F(3,63) = 7.104, p<.001, resp.) (Figure 4).

## Discussion

In this study we used the balanced placebo design [21] to investigate the effects of ginger and of the information on the drug received on symptoms of motion sickness (SR), and associated behavioral (HM, RT), and specific (EGG) and unspecific (cortisol) physiological functions in a rotation chair paradigm. Because in previous studies we had observed substantial sex differences on the subjective response [17], [18], we balanced the experimental groups for participant’s sex. To control for experimenter effects, groups and participant’s sex were balanced for the two experimenters and they were included in post-hoc analyses. Possible confounding variables were experiences with motion sickness (MSSQ), expectancies about own susceptibility to nausea and about ginger effects, and baseline cortisol levels.

Different from previously published work [1], [3]–[5], ginger had no antiemetic effect on any of the outcome measures in our rotation chair procedure. Also, the ginger information per se was not effective in preventing or reducing motion sickness or in affecting behavioral and physiological measures. These results seem to be stable since they were not influenced by any of our covariates such as previous experiences with motion sickness (as measured by the MSSQ), by the expectancy of ginger effects, or by salivary cortisol levels.

The missing effect of ginger may be explained by the higher intensity of stimulation that we used in this study (5×2 minutes instead of 5×1 minute as in most previous studies) and that may have been too strong to be overcome by a weak antiemetic.

Furthermore, suggestions did not affect the outcome measures. Participants were informed about the antiemetic effects of ginger in the informed consent form, and the suggestion to have received ginger (or placebo) was provided by a short sentence written on a piece of paper that participants found in an envelope directly prior to the rotation procedure. At this point of time, they possibly did not remember the whole information about the effects of ginger, the suggestion maybe was too brief, and there was not enough time to develop positive expectations before rotations started.

So far, our results agree to the study by Levine et al. [20] as they did not find any differences between their control group which were told they received a placebo and their positive-expectancy group which were told they received an antiemetic pill. Unfortunately, they did not report analysis of the sex of participants or experimenter(s).

However, a few published studies have shown significant sex differences in the susceptibility to nausea [14] as well as in the placebo and nocebo response both in nausea [17], [18], and pain [19], [28]. We therefore included the sex of participants in our analysis but group differences remained insignificant.

To control for experimenter effects, participants were randomly and balanced assigned to two experimenters (AM, JS). Post-hoc analyses of experimenter effects revealed a highly significant interaction with drug, information, and participant’s sex for behavioral measures in men only. When male participants received ginger there were no effects of information or experimenter on head movements, but when they received placebo they were susceptible to an interactional effect of information given and experimenter: They performed more head movements with the ginger information provided by the female experimenter and with the placebo information provided by the male experimenter.

Such complex interactions have not yet been described in the literature about nausea and have to be taken with great care, as we did not systematically vary the experimenters but employed only two (of different sex): The effect therefore might as well represent “personality” of the two experimenters rather than their sex. Furthermore, this was a post-hoc analysis to explore further mechanisms in expectation-induced placebo effects in nausea and sample sizes became small. In the light of its implications for further experimental and clinical studies, however, these results are of interest.

This complex interaction comprises two important issues in placebo research: Firstly, the question whether drug and placebo effects are additive or not [22], and secondly, sex differences in expectancy induced placebo effects and psychosocial interactions with experimenters, e.g. [18], [28], [29]. Kirsch [22] raised the question whether placebo effects in drug groups are as high as in placebo groups in clinical trials and suggested the balanced placebo design for further investigation as it allows separating the true drug effect from the placebo effect. Balanced placebo design studies with everyday-drugs like nicotine showed that expectancy effects more often occurred in placebo groups than in “drug” groups, e.g. [30], and a recent study with an analgetic revealed that this is particularly true for male participants [31]. In our study expectancy effects in men did not occur when ginger was given and we saw a small effect of ginger in the electrogastrogram in men only. As far as known, no differences in the pharmacological effect of ginger between women and men have been found by now. Another explanation could be that women’s behavior is stronger connected to their symptoms than men’s behavior as the found correlations would indicate.

Differences between female and male patients or participants are often described as “gender” effects in studies but psychometric assessment of gender have rarely been conducted, and physiological appearance of participants or self-reports of sex are used. Gender and sex differences in pain perception and processing are well documented in the literature and are assumed to be associated to gender role socialization [32]. A similar interactional effect of participant’s and experimenter’s sex on behavioral but not on subjective measures – like in our study – was found in a study comparing effects of sex and status of experimenters with a ice-water test by employing two experimenters for each condition [33]: Male participants could place their hand longer in ice-water with a female compared with a male experimenter, and female participants could tolerate ice-water longer with a male experimenter compared with a female experimenter, but there was no such interaction for subjective pain intensity [33].

Published data on sex differences in placebo and nocebo responses are rare and in part controversial, as we have shown in a review recently [29], and this refers to both experimental and clinical data. We concluded that sex differences in the placebo response occur predominantly because of psychosocial interactions between participants and experimenters, and not because of physiological differences between women and men [29].

Flaten et al. [19] were the first to describe that placebo analgesia may as well be affected by the sex of the participants: In their ischemic pain paradigm, only males responded to the suggestion of pain decrease during a placebo analgesia procedure [19], but in this case all experimenters were females. To explore the relationship between placebo analgesia and sex of participants and experimenters further, they employed three male and three female experimenters to study placebo analgesia in healthy participants, half males and females [28]. They again found reduced (heat) pain reporting in male participants only when the experimenters were females, and this placebo analgesia was not associated with changes in autonomic activity allowing them to conclude that the observed gender/sex effects on placebo analgesia may be mediated by psychosocial factors. This was further supported by a study [34] showing that the placebo analgesia response seen in men only was mediated by anticipatory stress response that was stronger in men than in women when expecting placebo analgesia. In contrast, another study by the same group [35] with four female and four male experimenters showed that male participants reported higher placebo analgesia to male experimenters compared with female experimenters. Interestingly and in line with studies about pain perception in general [32], [33], male participants reported lower overall pain to female experimenters than to male experimenters. The authors concluded that the experimenter’s sex might not be systematically related to placebo analgesia but maybe his/her behavior [35]. However, these studies consistently revealed that male participants were more prone to placebo effects by suggestions than women as it has also been shown for nausea in a previous study from our group [18].

These studies allow concluding that the results of our study reported here may be due to complex psychosocial factors rather than to “simple” sex differences. This is further supported by the fact that behaviors (RT, HM) rather than subjective symptom reports (SR) appear to be sensitive to such psychosocial modulation.

An explanation for higher susceptibility to suggestions in men may originate in classical gender role socialization whereby men intend to impress women with their behavior [33], and are often socially punished by their male peers when deviating from “tough” male behaviors [32]. In case of symptoms they are willing to endure a situation in general, and especially when additional symptom reduction is suggested. This furthermore could explain the discrepancy between effects in behavioral and subjective measures. Whether this effect is more pronounced with a female or a male experimenter is probably due to personality of the actual experimenter or due to specific participant-experimenter interactions. This hypothesis still has to be investigated in further studies.

Specific and unspecific physiological measures – gastric myoelectrical activity and salivary cortisol – appear neither be sensitive to detect such social interactions that were found for behavioral measures, at least not with our experimental paradigm. The results however show – as predicted – a decrease in normal gastric activity and an increase in tachygastria with rotation onset and even before, as described in the literature [8], [27], [36]. We also found a moderate effect of ginger in slowing this ratio-shift towards tachygastria in anticipation of the rotation, although the shift ultimately occurred in a similar manner as placebo in response to the rotation procedure. Again this may have been due to the intensity of the stimulus applied.

A few other limitations of our study need to be acknowledged. One is that participants were appointed to the laboratory either at 8.00 or at 10.30 a.m., and significantly different cortisol levels at these two time points could not only explain the differences in baseline expectancy levels but also subsequent nausea experience, as we have shown in another experiment [36]. However, controlling for saliva cortisol levels had no influence on results but balancing groups for the time of investigation may be a prerequisite for future studies. Another limitation is that our sample size has only been sufficient to detect a large but not a moderate effect in the balanced placebo design. Furthermore, we did not intend to study gender/sex differences and experimenter effects, not to speak of their gender/sex. Whereas post-hoc calculation revealed a sufficient sample size to detect a moderate effect in the four-factorial MANOVA (analysis with the software G*power: Cohen’s f^2^ = .15, alpha = .05, and power = .95 revealed a total n of 62), small cells made the results less robust and difficult to generalize. Therefore, our data must be taken as preliminary and providing first evidence for a complex social interaction between the gender/sex of both experimenters and participants when it comes to placebo (and nocebo) responses in experimental nausea. As social interactions between participants and experimenters could affect every placebo response and therefore occur in nearly every experimental and clinic trial, further investigations are of importance.

We here used the so-called “balanced placebo design” [21] that has been proposed to better separate drug effects from placebo effects than conventional double-blinded randomized placebo-controlled drug trials as it allows to separate the “true” drug effect (drug given but told to have received placebo) from the compound “drug plus placebo” effect when chances to receive the drug are 50% [37]. The “additive model” that is underlying all current drug testing has received increasing criticism [22]. In addition to its deceptive nature [38] that is inherent to all placebo research, its major disadvantage is the fact that participants are informed about the drug they have received prior to the testing of its effects, and this may cause mistrust and irritation in usually well-informed human participants, such as students at medical schools. We have recently [39] proposed an alternative design for this purpose (called the balanced cross-over design) to prevent such bias in data collection that can neither be ruled out nor appropriately controlled for otherwise. However, while this model requires independent validation, the balanced placebo design was successfully employed in the current study to uncover complex interactions of the sex of participants and experimenters on behavior outcomes.
